# Podocytes from hypertensive and obese mice acquire an inflammatory, senescent, and aged phenotype

**DOI:** 10.1152/ajprenal.00417.2023

**Published:** 2024-02-29

**Authors:** Sierra R. McKinzie, Natalya Kaverina, Robert Allen Schweickart, Christopher P. Chaney, Diana G. Eng, Beatriz Maria Veloso Pereira, Bryan Kestenbaum, Jeffrey W. Pippin, Thomas Carroll, Oliver Wessely, Stuart J. Shankland

**Affiliations:** ^1^Division of Nephrology, Department of Medicine, University of Washington, Seattle, Washington, United States; ^2^Lerner Research Institute, Cleveland Clinic Foundation, Cleveland, Ohio, United States; ^3^Department of Medicine, University of Texas Southwestern Medical Center, Dallas, Texas, United States; ^4^Department of Physiology and Biophysics, University of São Paulo, São Paulo, Brazil

**Keywords:** aging, chronic kidney disease, hypertension, obesity, senescence

## Abstract

Patients with hypertension or obesity can develop glomerular dysfunction characterized by injury and depletion of podocytes. To better understand the molecular processes involved, young mice were treated with either deoxycorticosterone acetate (DOCA) or fed a high-fat diet (HFD) to induce hypertension or obesity, respectively. The transcriptional changes associated with these phenotypes were measured by unbiased bulk mRNA sequencing of isolated podocytes from experimental models and their respective controls. Key findings were validated by immunostaining. In addition to a decrease in canonical proteins and reduced podocyte number, podocytes from both hypertensive and obese mice exhibited a sterile inflammatory phenotype characterized by increases in NLR family pyrin domain containing 3 (NLRP3) inflammasome, protein cell death-1, and Toll-like receptor pathways. Finally, although the mice were young, podocytes in both models exhibited increased expression of senescence and aging genes, including genes consistent with a senescence-associated secretory phenotype. However, there were differences between the hypertension- and obesity-associated senescence phenotypes. Both show stress-induced podocyte senescence characterized by increased* p21* and *p53*. Moreover, in hypertensive mice, this is superimposed upon age-associated podocyte senescence characterized by increased* p16* and *p19*. These results suggest that senescence, aging, and inflammation are critical aspects of the podocyte phenotype in experimental hypertension and obesity in mice.

**NEW & NOTEWORTHY** Hypertension and obesity can lead to glomerular dysfunction in patients, causing podocyte injury and depletion. Here, young mice given deoxycorticosterone acetate or a high-fat diet to induce hypertension or obesity, respectively. mRNA sequencing of isolated podocytes showed transcriptional changes consistent with senescence, a senescent-associated secretory phenotype, and aging, which was confirmed by immunostaining. Ongoing studies are determining the mechanistic roles of the accelerated aging podocyte phenotype in experimental hypertension and obesity.

## Graphical abstract

**Figure:**
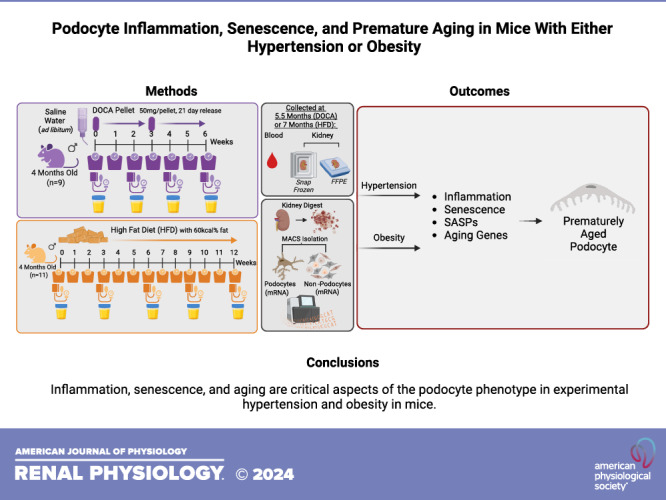

## INTRODUCTION

The incidence and prevalence of hypertension (1) and obesity (2) are increasing globally. Both are independent risk factors for cardiovascular disease and have major negative impacts on the kidney. Hypertension is considered the second leading cause of chronic kidney disease (3). The underlying mechanism(s) are likely multifactorial but include impairment of the glomerular autoregulatory mechanisms that normally protect the glomerulus from pathological alterations in systemic blood pressure (4–6). Several clinical studies show that hypertension contributes to podocyte injury. Wang et al. (7) showed that the number of podocytes per glomerulus was lower in patients with biopsy-proven hypertensive nephrosclerosis and that the patient’s urines exhibited higher levels of podocyte markers than those of healthy controls, suggesting active podocyte shedding. Naik et al. (8) demonstrated that urinary podocyte markers correlate with systemic blood pressure and that podocyte loss precedes apparent kidney disease in subjects with hypertension. The latter was confirmed in patients with hypertensive nondiabetes (9). Finally, examining autopsy samples and living kidney donors, lower podocyte density was also observed in subjects with hypertension without apparent kidney disease (10, 11). Animal models have validated these clinical reports. For example, podocyte injury in the deoxycorticosterone acetate (DOCA) hypertensive model in rats and mice has shown roles for TRPC5 (12) and claudin-5 (13), whereas deacetylation of Septin4 mitigated podocyte damage in hypertensive mice (14).

A similar detrimental effect has been observed for obesity, which is associated with low-grade albuminuria and a decline in kidney function (reviewed in Ref. 15). Like hypertension, changes to podocytes occur in obesity and obesity-related glomerulopathy. These include podocyte effacement, enlargement of foot processes, detachment, and reduced density (15). In addition, the podocyte transcriptome changes are characterized by the nascent expression of mesenchymal markers. Finally, patients with obesity-related glomerulopathy can lose up to 45% of their podocytes (16). Obesity leads to progressive increases in the glomerular filtration rate, renal plasma flow, and filtration fraction, synonymous with glomerular hyperfiltration. The relationship between obesity and kidney impairment was further reinforced in patients who lost weight following bariatric surgery with subsequent reduced glomerular hemodynamics (17). Mechanistically, reduced levels of adiponectin and leptin, together with the development of insulin resistance and hypercholesterolemia are some of the pathways that contribute to the decline in kidney function due to obesity (18) (reviewed in Ref. 19). A subset of patients with obesity develop more severe kidney damage due to obesity-related glomerulopathy, which is a distinct form of peri-hilar focal segmental glomerulosclerosis (FSGS) with higher levels of proteinuria but are typically not in the nephrotic range (15, 19). Obesity-related podocyte injury has been reproduced in experimental rodent studies using high-fat diet (HFD) (20–23). For example, obese mice develop podocyte damage, including increased foot process diameter, swollen and fused foot processes, and mitochondrial vacuolar degeneration (21, 22). These aberrant phenotypic changes were reduced upon treatment with the glucagon-like peptide-1 receptor agonist liraglutide (21).

Despite advances in the understanding of podocyte damage in hypertension and obesity, the pathways underlying these changes are not fully elucidated. To close these knowledge gaps by identifying new pathways responsible for podocyte injury in hypertension and obesity, we undertook an unbiased approach, performing bulk mRNA sequencing of podocytes isolated from representative hypertensive (DOCA-treated) and obese [high-fat diet (HFD)-fed] mouse models. The data demonstrate that either hypertension or obesity in mice resulted in podocytes acquiring an inflammatory, senescent, and aged phenotype.

## METHODS

### Mice and Experimental Models

All mice were housed in the animal care facility at the University of Washington under specific pathogen-free (SPF) conditions. Mature 4-mo-old adult male C57BL/6J mice were purchased from The Jackson Laboratory (Stock No: 000664, Bar Harbor, ME) and allowed to acclimate for at least 1 wk in our facilities before starting experiments or being euthanized. All animals were co-housed in social groups of two to five animals, provided with environmental enrichment (nestlets), maintained on a 14:10-h light-dark cycle, at 70 ± 5°F and 50 ± 10% relative humidity, with ad libitum access to food and water. Mice were euthanized at specified time points by cervical dislocation according to American Veterinary Medical Association (AVMA) guidelines for the euthanasia of animals by certified personnel, and tissues were processed for RNA and histology. To address the Reduction aspect of the 3Rs (Animal Use Alternatives), we have a bank of aged male C57BL/6J mice, also obtained from The Jackson Laboratory, aged and euthanized at various time points for use as controls. Blood pressure was measured with the CODA 6 noninvasive tail-cuff system (Kent Scientific, Torrington, CT) as previously described (24–26). Pooled spot urines were collected on the specified collection days. Animal protocols were approved (2968-04) by the University of Washington Institutional Animal Care and Use Committee.

### Deoxycorticosterone Acetate Salt Model

**Figure 1.**
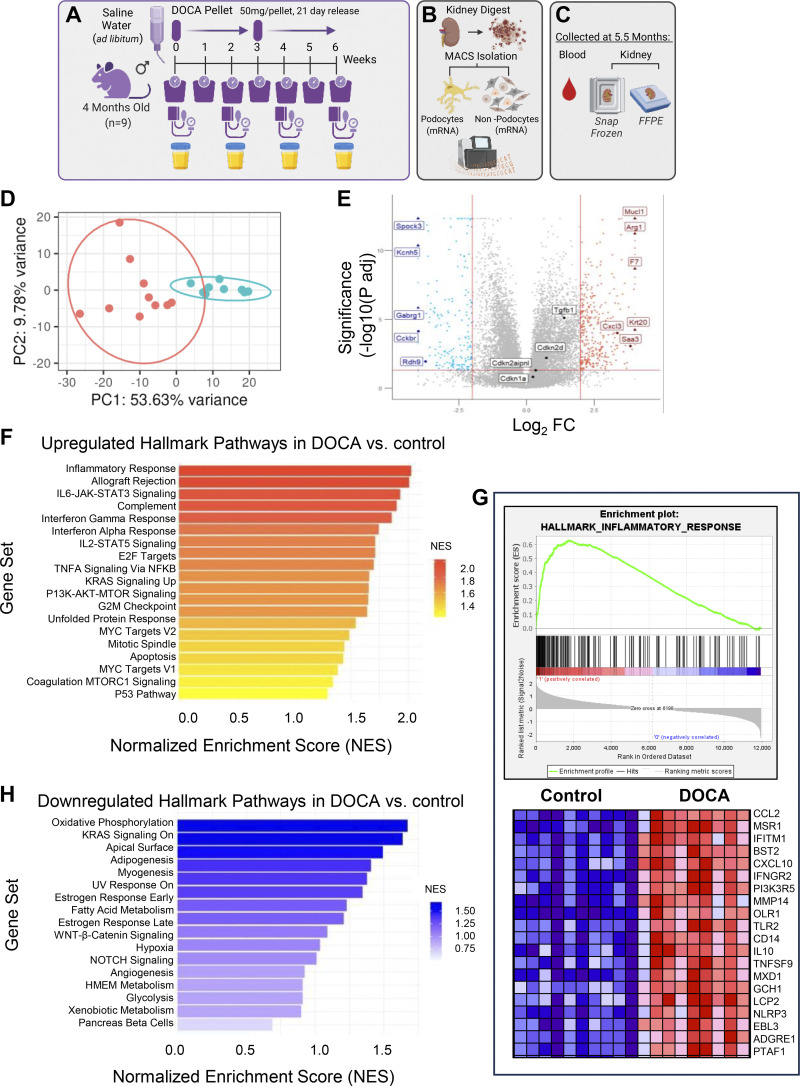
Transcriptomic changes in podocytes measured by RNA-sequencing from deoxycorticosterone acetate (DOCA)-treated mice. *A*–*C*: schematic of the experimental design. Following 6 wk of DOCA treatment (*A*), podocytes were isolated by magnetic-activated cell sorting (MACS) and underwent bulk RNA-sequencing (*B*), and kidneys were also collected and fixed for immunostaining (*C*). *D*: principal component analysis (PCA). PCA shows clear separation of the DOCA-treated mice (blue color) from the control mice (red color). *E*: volcano plot shows separation of individual genes that are increased (red), decreased (blue), or unchanged (gray) in hypertensive mice compared with controls. *F*–*H*: Hallmark pathways. *F*: Hallmark gene sets of the highest pathways enriched by Gene Set Enrichment Analysis (GSEA) show a predominance of inflammatory pathways in DOCA-treated mice. *G*: the top 20 genes in the inflammatory response pathway are shown. *H*: GSEA Hallmark pathways downregulated in podocytes upon DOCA treatment shows that oxidative phosphorylation was the most dysregulated Hallmark pathway.

Four-month-old mice (*n* = 9) were given two sequential subcutaneous deoxycorticosterone acetate salt (DOCA) pellet implants (50 mg/pellet, 21-day release, Innovative Research of America, Sarasota, FL) 3 wk apart for a total of 6 wk of sustained DOCA release (Fig. 1*A*). For the 6-wk duration of the experiment, animals received supplemental ad libitum saline water for drinking and their regular house water. Animals were weighed weekly, and urine and blood pressure were collected every 2 wk. At the end of 6 wk, animals were euthanized and processed as aforementioned (Fig. 1, *B* and *C*). Kidneys from mice aged 4 (*n* = 5) and 5.5 (*n* = 5) mo of age served as baseline and normal controls, respectively.

### High-Fat Diet-Induced Obesity Model

Four-month-old mice (*n* = 11) were provided ad libitum high-fat diet (HFD) with 60 kcal% fat (D12492, Research Diets Inc., New Brunswick, NJ) for 12 wk, which changed weekly. Weights were taken weekly for the duration of the study, whereas blood pressure and urine were taken every 3 wk. At the end of 12 wk, animals were euthanized and processed as aforementioned (Fig. 4, *A–C*). Kidneys were taken from mice at 4 (*n* = 5) and 7 (*n* = 5) mo of age that were maintained on house chow with 13 kcal% fat (LabDiet 5053, Purina Mills, Gray Summit, MO) and served as baseline and normal controls.

### Magnetic-Activated Cell Sorting of Podocytes

Kidney tissue (w/o the kidney capsule and surrounding fat) was placed into ice-cold RPMI 1640 medium (w/o l-glutamine and phenol red, GE Healthcare Bio-Sciences, Pittsburgh, PA) (Figs. 1*B* and 4*B*). After removal of the medulla, the remaining cortex was minced into fine pieces and digested in 0.2 mg/mL Liberase TL (Sigma Aldrich, St. Louis, MO), 100 U/mL DNAse I (Sigma Aldrich, St. Louis, MO) in RPMI 1640 medium (w/o l-glutamine and phenol red) by shaking at 37°C for 30 min. The digest was passed through an 18-G needle (Becton Dickenson, Franklin Lakes, NJ) 10 times. Enzymes were inactivated by adding 5 mL of RPMI 1640 medium (w/o l-glutamine and phenol red) supplemented with 1 mM sodium pyruvate (Thermo Fisher Scientific, Waltham, MA), 9% Nu-Serum IV Growth Medium Supplement (Corning Incorporated—Life Sciences, Durham, NC), and 100 U/mL penicillin-streptomycin (Thermo Fisher Scientific, Waltham, MA). The cell suspension was passed through a 100-µm and a 40-µm cell strainer (BD Biosciences, San Jose, CA) and pelleted by centrifugation at 200 *g* at 4°C for 5 min. Cells were resuspended in media containing two rabbit anti-nephrin antibodies (1:100, Abcam, Cambridge, MA). After 1 h at 4°C, cells were pelleted, washed in media, and incubated with anti-rabbit microbeads (Miltenyi Biotec, Auburn, CA) along with Alexa Fluor 594-conjugated AffiniPure donkey anti-rabbit IgG 1:200 (to visualize binding of the nephrin antibodies to the podocytes) for 30 min at 4°C. Cells were pelleted and washed in PBS with 0.5% BSA and 2 mM EDTA and applied to magnetic-activated cell sorting (MACS) LS columns (Miltenyi Biotec, Auburn, CA) to separate microbead-bound podocytes from the other kidney cells gently. Cells eluted through the magnetic field were collected, pelleted, and designated nonpodocyte (NP) cell fractions. LS columns were removed from the magnetic field and then washed with PBS with 0.5% BSA and 2 mM EDTA, and podocytes were collected. A small aliquot of each fraction was imaged using an EVOS FL Cell Imaging System to verify podocyte isolation based on the presence of a nephrin antibody. In addition, qRT-PCR for a panel of podocyte genes was performed in both podocyte and nonpodocyte fractions to confirm cell type identity (27).

### RNA Isolation, qRT-PCR, and Library Preparation and Sequencing

Messenger RNA was isolated using the RNeasy Mini Kit (Qiagen, Germantown, MD) as per the manufacturer’s instructions and used for bulk mRNA sequencing or converted to cDNA by reverse transcription with the High-Capacity RNA-to-cDNA Kit (Thermo Fisher, Waltham, MA) and used for quantitative real-time PCR analysis. qRT-PCR was performed using iTaq SYBR Green Supermix (Bio-Rad, Hercules, CA), QuantiTect Primer Assay (Qiagen, Germantown, MD) for *Cdkn2a* (p16 INK4A variant 2 QT01164898, p19ARF variant 1 QT01164891) and *Ckn1a* (p21 QT01752562) on the QuantStudio 6 Flex real-time PCR System (Applied Biosystems) as we have previously described (27). Relative mRNA expression levels were normalized to glyceraldehyde-3-phosphate dehydrogenase (*Gapdh*). Psomagen, Inc. (Rockville, MD) performed library generation and bulk next-generation mRNA sequencing using TruSeq RNA Library Prep Kits (Illumina, San Diego, CA) and the Illumina platform. The transcriptomic data were submitted to the NCBI’s Gene Expression Omnibus (GEO) sequence repository and are available under GEO Series Accession No. GSE255723.

### Data Analysis: Alignment and Quantification

Transcript abundance was estimated without aligning reads using Salmon (28) against an index of coding sequences from the Ensembl GRCm38 assembly. Transcript-level abundance was imported and count and offset matrices generated using the tximport R/Bioconductor package (29). Differential expression analysis was performed using the DESeq2 R/Bioconductor package (30). Principal component analysis (PCA) was performed using pcaExplorer (v2.26.1), considering the top 500 genes (31). Note that samples with low read counts did not cluster with the other samples and were flagged as outliers and removed. Gene Set Enrichment Analysis (GSEA) was performed using the Molecular Signature Database (MSigDB) as described (32). GSEA bar graphs were created in R (v.4.3.1) using GGplot2 (v.3.4.2), Dplyr (v.1.1.2), and Tidyverse (v.2.0.0).

### Immunostaining

Immunoperoxidase staining was performed on 4-µm thick formalin-fixed paraffin-embedded (FFPE) mouse and human kidney sections as previously described (33) (Figs. 1*C* and 4*C*). Briefly, paraffin-embedded, formalin-fixed sections were incubated in Histoclear (National Diagnostics, Atlanta, GA) and graded a series of ethanol for rehydration. Sections were boiled in antigen retrieval buffer (citric acid buffer pH 6.0 or EDTA buffer pH 6.0, pH 8.0). Background Buster (Accurate Chemical & Scientific, Westbury, NY), goat anti-rabbit Fab fragment, and rabbit Fab fragment (Jackson ImmunoResearch Laboratories, West Grove, PA) were used to avoid nonspecific protein binding. Endogenous biotin activity was suppressed using an Avidin/Biotin Blocking Kit (Vector Laboratories, Burlingame, CA). Initial primary antibodies were incubated overnight at 4°C. In the case of double immunostaining, subsequent primary antibodies were incubated overnight at 4°C or for 3 h at room temperature. Secondary antibodies and streptavidin conjugates were incubated for 1 h at room temperature. The primary antibodies used in the study are summarized in Supplemental Table S1. Diaminobenzidine (Sigma Aldrich, St. Louis, MO), with or without 8% nickel chloride, was precipitated to visualize immunoperoxidase staining.

### Microscopy and Imaging

Kidney sections were analyzed under an EVOS FL Cell Imaging System (Life Technologies) using the ×20 objective. Quantification of p57, podocin, Wilms’ tumor 1 (WT-1), vascular endothelial growth factor A (VEGFa), synaptopodin, p16, p21, p19, and senescence-associated-β-galactosidase (SA-β-gal) staining was performed using ImageJ 1.46r software (National Institutes of Health). A minimum of 40 glomeruli were assessed for quantification.

Podocyte density was determined through p57 nuclear staining. The calculation involved counting the number of p57-positive cells in a single glomerulus and dividing it by the measured glomerular tuft area. The values were then adjusted by referring to Venkatareddy et al.’s (34) methods. The final podocyte density was expressed as podocytes per total tuft volume (pods/×10^6^ μm^3^). Once the podocyte densities were calculated, the values were averaged (34).

### SA-β-Galactosidase Activity Staining

Frozen sections (Figs. 1*C* and 4*C*, 10 μm thick) were warmed from −80°C to room temperature and allowed to air dry. Sections were then treated in accordance with the manufacturer’s protocol for the SA-β-gal staining kit (Cell Signaling Technology, 9860) as previously described (33).

### In Situ Hybridization

*Cdkn2a* (p16 INK4A, variant 2, and p19 ARF, variant 1) mRNA expression was determined by RNAscope 2.0 following Advanced Cell Diagnostics (ACD, Newark, CA) kit instructions. In brief, freshly cut 4-µm formalin-fixed paraffin-embedded (FFPE) (Figs. 1*C* and 4*C*) slides were baked for 1 h at 60°C. Samples were deparaffinized and pretreated. Target probes for *Cdkn2a* (p16 INK4A, variant 2, and p19 ARF, variant 1) were hybridized. The signal was amplified and developed. Sections were dehydrated through graded ethanol (50%, 70%, and 100%) and xylene and then mounted with Cytoseal XYL (Thermo Fisher Scientific Inc., Waltham, MA). Staining data were recorded according to the presence of punctate nuclear and cytoplasmic staining and signal intensity.

### Statistics

Statistical analysis was performed using GraphPad Prism 10.0 (GraphPad Software, Inc., San Diego, CA). *t* tests were performed when comparing only two groups of data, whereas repeated-measures one-way ANOVAs were performed when making multiple comparisons. A *P* value threshold of 0.05 was used to determine statistical significance and data are shown as the means ± SD. For the RNA-sequencing results, a *P* adjusted value threshold of 0.05 was used to determine statistical significance.

## RESULTS

### Acute Hypertension in Mice Is Accompanied by Senescence and a Senescent-Associated Secretory Phenotype in Podocytes

In control mice, the mean arterial blood pressure (MAP) increased by 3% from 4 to 5.5 mo of age (94 ± 12 vs. 97 ± 15 mmHg, *P* = 0.7162) (Supplemental Fig. S1*A*). To investigate the glomerular phenotype due to hypertension, a separate group of 4-mo-old mice were implanted with deoxycorticosterone acetate (DOCA) pellets and provided ad libitum access to saline water. Following the initiation of this regime, the MAP was assessed every other week, and the glomerular state was assessed at *week 6* by bulk mRNA sequencing and immunofluorescence/histology analyses (Fig. 1, *A–C*). The average MAP was 95 ± 20 mmHg at 4 mo of age in this group of mice pre-DOCA implantation. Thereafter, the MAP increased by 24% at 4.5 mo (118 ± 23 mmHg, *P* = 0.009), by 44% at 5.0 mo (137 ± 42 mmHg, *P* = 0.0001), and by 22% at 5.5 mo (116 ± 21 mmHg, *P* = 0.09) (Supplemental Fig. S1*B*). As expected, the changes in MAP were paralleled by a decrease in renin-positive cells in the DOCA-treated animals (Supplemental Fig. S1, *C*–*E*).

Podocytes were isolated and processed for bulk mRNA sequencing to obtain an unbiased view of the transcriptional changes caused by hypertension. PCA analysis and a volcano plot comparing the two conditions (control vs. DOCA) identified dramatic differences (Fig. 1, *D* and *E*). Gene Set Enrichment Analysis (GSEA) examining the Hallmark gene sets showed that the highest pathways enriched in podocytes from DOCA-treated mice were related to inflammation, such as those characterizing the inflammatory response, allograft rejection, IL6-JAK-Stat3, interferon α/γ IL2-Stat5, and TNFa signaling (Fig. 1*F*). A heatmap depicting expression of the top 20 genes in the inflammatory response Hallmark pathway is shown in Fig. 1*G*. We recently reported that specific inflammatory pathways, i.e., programmed cell death protein 1 (PD-1) and NLR family pyrin domain containing 3 (NLRP3) inflammasome signaling, contribute to injury in middle-aged and aged podocytes (33, 35). In fact, the expression of the transcripts for programmed death ligand 1 (*Cd274*) and programmed death ligand 2 (*Pdc1lg2*) were increased 2.1- and 1.8-fold, respectively, in podocytes from DOCA-treated mice compared with control mice. Similarly, NLRP3 inflammasome pathway components, including *Nlrp3* and *Casp1,* were increased by RNA and protein levels (Supplemental Fig. 2, *A*–*G*). Finally, upstream regulators of the NLRP3 inflammasome, Toll-like receptors (TLRs) such as* Tlr4* (36), were increased in podocytes from DOCA-treated mice (Supplemental Fig. S2*H*). Together, these data demonstrate that podocytes from hypertensive mice acquire an extensive inflammatory phenotype.

### Reduced Hallmark Pathways in DOCA-Treated Mice

Next, we investigated the GSEA pathways downregulated in podocytes upon DOCA treatment (Fig. 1*H*). Among these, oxidative phosphorylation was the most dysregulated Hallmark pathway with *Uqcrh* (ubiquinol-cytochrome *c* reductase hinge protein, 1.53-fold downregulated) and *Ndufb*3 (NADH: ubiquinone oxidoreductase subunit B3, 1.32-fold downregulated). Other notably downregulated pathways were Kirsten rat sarcoma virus (KRAS) signaling and apical surface signaling. The latter includes genes such as EPH receptor B4 (*Ephb4*, 2.0-fold downregulated), a signaling pathway reported to allow podocytes to survive transient capillary collapse during glomerular disease (37), actin filament-associated protein 1-like 2 (*Afap1l*2, 1.6-fold downregulated), ephrin A5 (*Efna5*, 2.3-fold downregulated), and GATA binding protein 3 (*Gata3*, 2.7-fold downregulated). Finally, the Wnt/β-catenin signaling Hallmark pathway, which includes genes like *Jag1, Notch4, Hey2, Jag2, Fzd8, Hdac11, Dll1*, and *Gnai1*, which are known to regulate several key downstream events required for normal podocyte function (38, 39), was downregulated. Together, these data show that several pathways and individual genes important for podocyte homeostasis were lowered in podocytes from hypertensive mice due to DOCA administration.

### Podocytes Express Aging Genes in DOCA-Treated Mice

Activating inflammatory pathways such as PD-1 and NLRP3 inflammasome signaling is part of healthy podocyte aging (33, 35). We thus analyzed genes considered to represent an aged phenotype, i.e., the RODWELL_AGING_KIDNEY_UP gene list (40) (Table 1). Among the genes listed, many were statistically significantly increased in the podocytes of DOCA-treated mice. Examples included *Mmp7* (27-fold higher), *Fn1* (10.7-fold higher), *Timp1* (10.2-fold higher), *Vcam1* (4.7-fold higher), *Lmnb1* (3.0-fold higher), *Ccl2* (4.4-fold higher), *Serpine1* (2.2-fold higher), and *Nfkb1* (1.9-fold higher). Importantly, validation of the increase in *Timp1* (Supplemental Fig. S3, *A*–*C*), *Lmnb1* (Supplemental Fig. S3, *D*–*F*), *Nfkb1* (Supplemental Fig. S3, *G*–*I*), *Vcam1* (Supplemental Fig. S3, *J*–*L*), *Mmp2* (Supplemental Fig. S3, *M*–*O*), and *Tgfb1* (Supplemental Fig. S3, *P*–*R*) were confirmed at the protein level by immunostaining.

**Table 1.** Rodwell_Aging_Kidney_Up in podocytes of DOCA mice vs. control mice

RODWELL_AGING_KIDNEY UP Genes		RODWELL_AGING_KIDNEY DOWN Genes	
Gene Name	*Fold Change Higher in DOCA vs. Control	Gene Name	*Fold Change Lower in DOCA vs. Control
*Mmp7*	27.4	*Adra2a*	−3.4
*Havcr1*	22.5	*Cdo1*	−3.0
*Gpnmb*	19.4	*Spon2*	−2.9
*Fn1*	10.7	*Fzd3*	−2.9
*Alpk2*	10.3	*Gabre*	−2.5
*Timp1*	10.2	*Nr2f1*	−2.5
*Col3a1*	9.4	*Rarres1*	−2.4
*Adam8*	7.2	*Tesc*	−2.3
*Vcan*	7.1	*Lix1*	−2.3
*Inhba*	7.0	*Erich3*	−2.2
*Msr1*	6.6	*Gfra1*	−2.2
*Col1a1*	6.4	*Tspan18*	−2.2
*C7*	5.6	*Gpx8*	−2.0
*Mrc1*	5.2	*Vtcn1*	−2.0
*Ms4a7*	5.1	*Tspan1*	−2.0
*Vcam1*	4.8	*Slc1a3*	−2.0
*Ccl2*	4.4	*Rbms3*	−2.0
*Col14a1*	4.3	*Kcnd3*	−2.0
*Slc15a3*	4.3	*Rflnb*	−1.9
*Clec7a*	4.2	*Prickle1*	−1.9
*Ccr1*	4.0	*Slc18a2*	−1.9
*Csf1r*	3.9	*Adgrg1*	−1.8
*C1qc*	3.9	*Aebp1*	−1.8
*Mpeg1*	3.9	*Cdh6*	−1.8
*C1qa*	3.9	*Wfdc2*	−1.8
*Cd14*	3.8	*Arvcf*	−1.7
*Nnmt*	3.7	*Shank2*	−1.7
*Lcp2*	3.7	*Itga1*	−1.7
*Rad54l*	3.6	*Pam*	−1.6
*Myo1f*	3.6	*Asns*	−1.6
*Tgfbi*	3.6	*Rab34*	−1.6
*Hgf*	3.6	*Nt5e*	−1.5
*Jchain*	3.5	*Pxdn*	−1.5
*Gbp5*	3.5	*Prom1*	−1.5
*C1qb*	3.5	*Mcam*	−1.5
*Cxcl14*	3.5		
*Cx3cr1*	3.4		
*Itgb2*	3.4		
*Rac2*	3.3		
*Lacc1*	3.3		
*Axl*	3.3		
*Ncf1*	3.2		
*Fcer1g*	3.2		
*Tbxas1*	3.2		
*Laptm5*	3.2		
*Lst1*	3.2		
*Grn*	3.2		
*Stab1*	3.2		
*Tyrobp*	3.1		
*Slamf8*	3.1		
*Rgs10*	3.1		
*Pik3cg*	3.1		
*Runx3*	3.1		
*Apbb1ip*	3.1		
*Dock2*	3.0		
*Gng2*	3.0		
*Ptpre*	3.0		
*Rgs1*	3.0		
*Milr1*	3.0		
*Arhgap30*	3.0		
*Evi2a*	3.0		
*Cxcl16*	2.9		
*Cxcl1*	2.9		
*Rnf213*	2.9		
*Parp14*	2.9		
*Celf2*	2.9		
*Arl4c*	2.9		
*Ifitm1*	2.8		
*Hcst*	2.8		
*Nlrc5*	2.8		
*Sting1*	2.8		
*Ptprc*	2.8		
*Vsir*	2.8		
*Loxl1*	2.8		
*Themis2*	2.7		
*Hcls1*	2.7		
*Zeb2*	2.7		
*Nfkbiz*	2.7		
*Calhm6*	2.7		
*Rab8b*	2.7		
*Pik3cd*	2.7		
*Cd53*	2.7		
*Myof*	2.7		
*Ikzf1*	2.7		
*Parvg*	2.6		
*Antxr2*	2.6		
*Prex1*	2.6		
*Samhd1*	2.6		
*Birc3*	2.6		
*Man2b1*	2.6		
*Cxcr4*	2.6		
*Trafd1*	2.6		
*Ppp1r18*	2.6		
*Selplg*	2.6		
*Fnbp1*	2.6		
*Igkc*	2.6		
*Arpc1b*	2.5		
*Cmtm3*	2.5		
*Arhgef6*	2.5		
*Coro7*	2.5		
*Il10ra*	2.5		
*Lyn*	2.5		
*Ehbp1l1*	2.5		
*Ptpn6*	2.5		
*Coro1a*	2.4		
*Akna*	2.3		
*Ifnar2*	2.3		
*Tap1*	2.3		
*Psmb8*	2.3		
*Casp1*	2.3		
*Jaml*	2.3		
*Psmb9*	2.3		
*Dtx3l*	2.2		
*Irf8*	2.2		
*Slc12a9*	2.2		
*Arhgap25*	2.2		
*Ccdc88a*	2.2		
*Dok3*	2.2		
*Marchf1*	2.1		
*Fxyd5*	2.1		
*Clec10a*	2.1		
*Calhm2*	2.1		
*Dok1*	2.1		
*Gmfg*	2.1		
*Stk10*	2.1		
*Srgn*	2.1		
*Vim*	2.1		
*Arhgdib*	2.1		
*Timp2*	2.1		
*Rps6ka1*	2.1		
*Rgs19*	2.1		
*Dock11*	2.1		
*Cybc1*	2.0		
*Pgm2l1*	2.0		
*Emp3*	2.0		
*Bhlhe41*	2.0		
*Mthfd1l*	2.0		
*Ggta1*	2.0		
*Ifnar1*	2.0		
*Rtn1*	2.0		
*Acer3*	2.0		
*Glipr2*	2.0		
*Irak2*	1.9		
*Rassf5*	1.9		
*Dkk3*	1.9		
*Rab31*	1.9		
*Ptpn22*	1.9		
*Trex1*	1.9		
*Rin3*	1.9		
*Arhgap45*	1.9		
*Tpm3*	1.8		
*Spon1*	1.8		
*Rnase6*	1.8		
*Plekho1*	1.8		
*Lpcat1*	1.8		
*Cmtm7*	1.8		
*Pald1*	1.8		
*Tmsb10*	1.8		
*St8sia4*	1.8		
*Plod3*	1.7		
*Pfn1*	1.7		
*Zcchc24*	1.7		
*Parp12*	1.7		
*Rftn1*	1.7		
*Stat6*	1.7		
*Coro1c*	1.7		
*Fkbp15*	1.7		
*Syngr2*	1.7		
*Reep4*	1.6		
*Itm2c*	1.6		
*Tbc1d2b*	1.6		
*Tapbp*	1.6		
*Rpl12*	1.6		
*Fhod1*	1.6		
*Clic1*	1.6		
*Rpl10*	1.6		
*Atp8b2*	1.6		
*Rpl27*	1.5		
*Ikbkb*	1.5		
*Mvp*	1.5		
*Crip1*	1.5		
*Cd47*	1.5		
*Ccm2*	1.5		
*Psme2*	1.5		
*Serping1*	1.5		
*Rnf166*	1.5		
*Il17ra*	1.5		
*Ppm1m*	1.5		

DOCA, deoxycorticosterone acetate. *Defined as >1.5-fold higher with adjusted *P* value <0.05.

**Figure 2.**
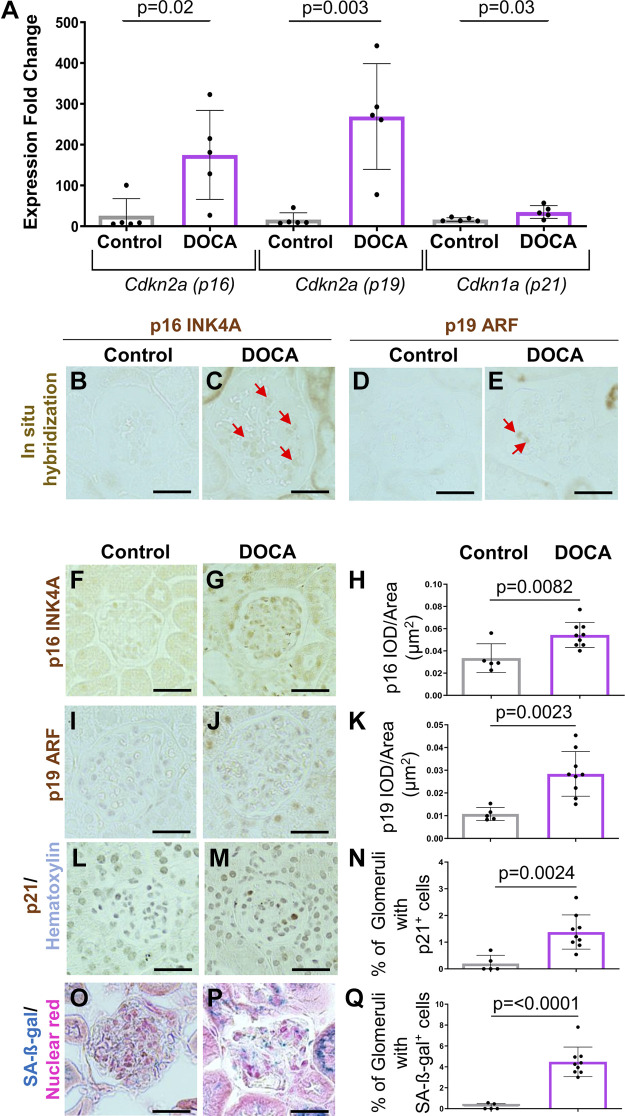
Age- and stress-associated senescent genes are increased in podocytes from young mice following deoxycorticosterone acetate (DOCA) treatment. *A*: qRT-PCR on isolated podocytes shows that the expression for *Cdkn2a* (p16 INK4A, variant 2), *Cdkn2a* (p19 ARF, variant 1), and *Cdkn1a* (p21) are higher in young DOCA-treated mice. *B*–*E*: in situ hybridization (ISH). ISH (brown color) was increased for *Cdkn2a* (p16 INK4A, variant 2) (*B* and *C*) and *Cdkn2a* (p19 ARF, variant 1) (*D* and *E*) in DOCA-treated mice. *F*–*N*: representative immunoperoxidase immunostaining (brown) and quantitation. Immunostaining validated the increases in p16 (*F*–*H*), p19 (*I*–*K*), and p21 (*L*–*N*). The senescent marker SA-β-galactosidase (SA-β-gal, blue) was higher in DOCA-treated mice (*O*–*Q*). Scale bar: 40 μm.

A similar trend was observed when transcripts for senescent-inducing genes, senescent markers, and the Reactome cellular senescence gene set were interrogated (Table 1). Compared with control mice, podocytes from DOCA-treated mice showed increased *Cdkn1a/*p21 (1.66-fold), *Cdkn2a/*p16 INK4A (5.36-fold), and *SerpinB2* (8-fold). qRT-PCR validates this for p16 INK4A (*Cdkn2a,* variant 2), p19 ARF (*Cdkn2a,* variant 1), and *Cdkn1a* (p21) (Fig. 2*A*) and by in situ hybridization for p16 INK4A (*Cdkn2a,* variant 2) and p19 ARF (*Cdkn2d*, variant 1) (Fig. 2, *B*–*E*) in mice given DOCA. Moreover, immunostaining showed elevated protein levels for p16 INK4A (*Cdkn2a,* variant 2), p19 ARF (*Cdkn2a,* variant 1), and p21 (*Cdkn1a*) in DOCA-treated mice compared with age-matched controls (Fig. 2, *F–N*). Finally, glomerular staining for the senescent marker SA-β-gal was higher in DOCA-treated mice than in age-matched control mice (Fig. 2, *O*–*Q*).

Like markers of senescence, analysis of genes/gene sets representing a senescent-associated secretory phenotype (SASP) were detected in DOCA-treated mice (Table 2). These included upregulated *Timp1* (10.2-fold) and *Tgfb1*(3.62-fold), which were also validated at the protein level by immunostaining (Supplemental Fig. S3, *A*–*C* and *P*–*R*). Analysis of genes of the SASP Reactome and the Fridman senescence genes were also enriched in podocytes of mice treated with DOCA (Table 3) and included ubiquitin-conjugating enzyme E2C (*Ube2c*, increased 6-fold) that decreases mitotic cyclins thereby limiting cell cycle progression (41) and interleukin 1A (*Il1a*, increased 5.6-fold), which increases in podocytes in human glomerulonephritis (42). Finally, we also observed the enrichment of gene sets of the *Tp53* and DNA repair pathways (Supplemental Fig. S4, *A*–*D*).

**Table 2.** Senescent-associated secretory phenotype in DOCA-treated mice

SASP Gene Set Identified in Ref. 27		Reactome_Senescence_Associated_Secretory_Phenotype_SASP			
		Up Genes		Down Genes	
Gene Name	*Fold Change	Gene Name	*Fold Change in DOCA vs. Control	Gene Name	*Fold Change in DOCA vs. Control
*Timp1*	10.2	*Ube2c*	6.1	*H3c15*	−1.8
*Igfbp2*	6.6	*Il1a*	5.6	*Igfbp7*	−1.6
*Ccl2*	4.4	*Cdkn2a*	5.4		
*Cxcl1*	3.0	*H4c11*	4.1		
*Irf5*	2.7	*Il6*	3.1		
*Mmp12*	2.6	*Ccna2*	2.9		
*Cebpb*	2.5	*Cebpb*	2.5		
*Timp2*	2.1	*Cdk6*	2.5		
*Ccl3*	2.0	*Rps6ka1*	2.1		
*Jdp2*	1.9	*Nfkb1*	1.9		
*Ccl5*	1.5	*H4c9*	1.9		
*Mmp13*	1.3	*Stat3*	1.8		
*Mmp2*	1.3	*Cdkn1a*	1.7		
*Igfbp3*	1.1	*Rela*	1.7		
*Hmgb1*	1.1	*Cdkn2c*	1.6		
*Igfbp4*	−1.1	*Cdk2*	1.5		
*Igfbp7*	−1.6				
*Igfbp5*	−2.6				
*Jchain*	3.5				

DOCA, deoxycorticosterone acetate; SASP, senescent-associated secretory phenotype. *Defined as >1.5-fold higher with adjusted *P* value <0.05.

**Table 3.** Senescent genes in podocytes of DOCA-treated mice

*Reactome_Cellular_Senescence*				*Fridman Senescence Genes*	
Up Genes		Down Genes			
Gene Name	*Fold Change in DOCA vs. Control	Gene Name	*Fold Change in DOCA vs. Control	Gene Name	*Fold Change in DOCA vs. Control
*Ube2c*	6.1	*Mapk10*	−3.9	*Fn1*	10.7
*Il1a*	5.6	*Id1*	−1.9	*Serpinb2*	8.1
*Cdkn2a*	5.4	*H3c15*	−1.9	*Igfbp2*	6.6
*H4c11*	4.1	*Mapk11*	−1.8	*Cdkn2a*	5.4
*Ccne1*	3.8	*Igfbp7*	−1.6	*Cxcl14*	3.5
*Ccne2*	3.4	*Cbx8*	−1.5	*Cd44*	3.4
*Il6*	3.1	*Tfdp2*	−1.5	*Tnfaip3*	3.3
*Lmnb1*	3.0			*Igfbp6*	2.8
*Ccna2*	2.9			*Irf7*	2.8
*Kdm6b*	2.8			*Irf5*	2.7
*Cebpb*	2.5			*Isg15*	2.3
*Cdk6*	2.5			*Stat1*	2.3
*Ets2*	2.4			*Thbs1*	2.3
*Ago3*	2.2			*Serpine1*	2.2
*E2f1*	2.2			*Vim*	2.1
*Rps6ka1*	2.1			*Cyp1b1*	1.9
*Nfkb1*	1.9			*Filip1l*	1.9
*Mapkapk2*	1.9			*Rab31*	1.9
*H4c9*	1.9			*Creg1*	1.9
*Mapkapk3*	1.8			*Cdkn1a*	1.7
*Mapk14*	1.8			*Rgl2*	1.6
*Stat3*	1.8			*Hps5*	1.5
*Tinf2*	1.7			*Mdm2*	1.5
*Ezh2*	1.7				
*Hmga1*	1.7				
*Ubn1*	1.7				
*Cdkn1a*	1.7				
*Rela*	1.7				
*Rad50*	1.7				
*Eed*	1.6				
*Cdkn2c*	1.6				
*Ep400*	1.5				
*Mdm2*	1.5				
*Ago4*	1.5				
*Cdk2*	1.5				
*Mdm4*	1.5				

DOCA, deoxycorticosterone acetate. *Defined as >1.5-fold higher with adjusted *P* value <0.05.

Together, these results show that podocytes from young mice with hypertension resulting from DOCA administration showed hallmarks for premature aging and the acquisition of a senescent and a SASP phenotype. Importantly, this includes the expression of aging-inducing senescent genes (p16 and p19), stress-inducing senescent genes (p21 and p53), and an enriched DNA repair pathway.

### Hypertension Decreases the Expression of Podocyte Canonical Genes and Podocyte Number

**Figure 3.**
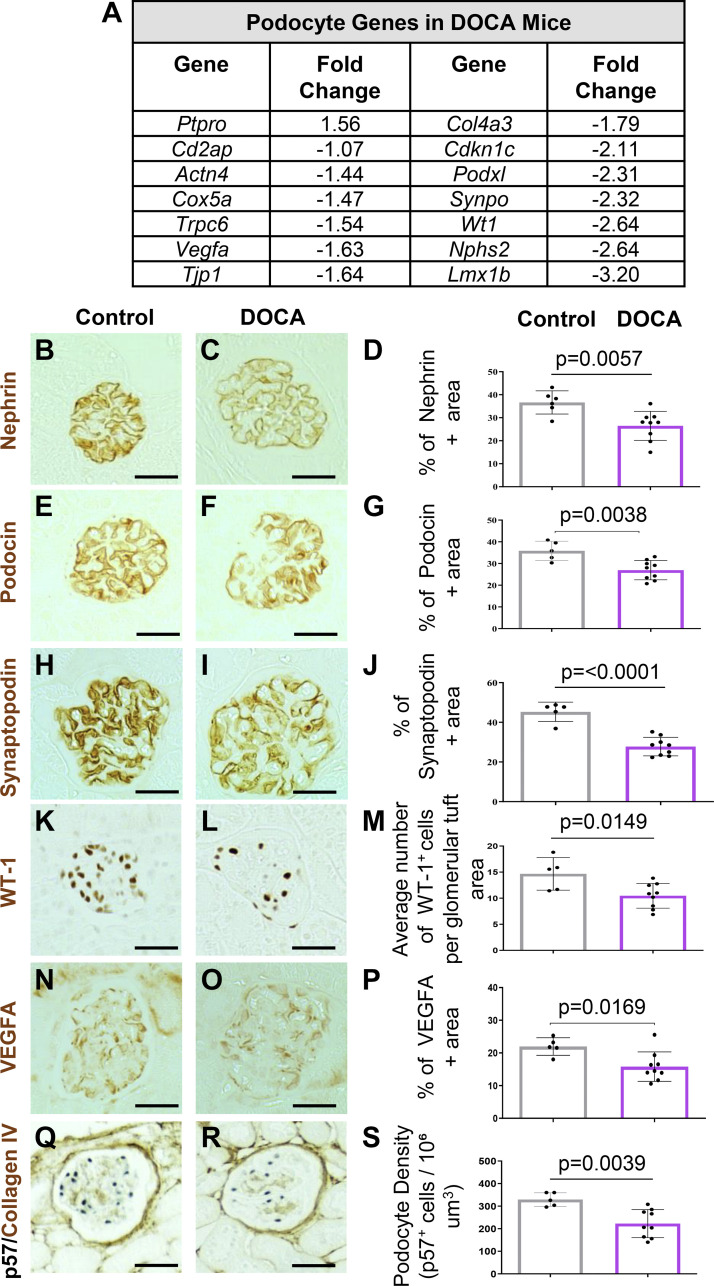
Deoxycorticosterone acetate (DOCA) treatment lowers the expression of canonical podocyte proteins. *A*: RNA sequencing results. The transcript levels for individual podocyte genes decreases in DOCA-treated mice compared with controls. *B*–*S*: representative immunoperoxidase immunostaining (brown) for individual podocyte proteins and accompanying graph showing their quantitation. In DOCA-treated mice, there was a decrease in immunostaining nephrin (*B*–*D*), podocin (*E*–*G*), synaptopodin (*H*–*J*), Wilms’ tumor 1 (WT-1; *K*–*M*), vascular endothelial growth factor A (VEGFa; *N*–*P*), and p57 (*Q*–*S*). Scale bar: 40 μm.

Besides the upregulation of inflammatory and senescent pathways, aging also results in a marked reduction of canonical podocyte genes (27, 33, 35, 43). Indeed, many canonical podocyte genes such as *Podxl*, *Synpo*, *Nphs2*, *Vegfa,* and *Tpj1* were downregulated in podocytes of DOCA-treated mice compared with controls (Fig. 3*A*). Interestingly, the podocyte gene *Ptpro/Glepp-1* was 1.6-fold higher in DOCA-treated mice, suggesting that injury may induce its expression (Fig. 3*A*). Importantly, these changes were confirmed by immunostaining for nephrin (Fig. 3, *B–D*), podocin (Fig. 3, *E–G*), synaptopodin (Fig. 3, *H–J*), Wilms’ tumor 1 (WT-1, Fig. 3, *K*–*M*), and vascular endothelial growth factor A (VEGFa, Fig. 3, *N*–*P*). Notably, these changes had physiological consequences as podocyte density, quantitated by immunostaining for the podocyte marker p57 (Fig. 3, *Q–S*) was significantly lower in DOCA-treated mice compared with control mice (329 vs. 223 p57^+^ cells/10^6^ μm^3^), *P* = 0.04). Finally, immunostaining for the endothelial cell marker ERG (Supplemental Fig. S4, *E* and *F*) and the activated parietal epithelial cell marker CD44 (Supplemental Fig. S4, *G* and *H*) did not change in mice given DOCA. These results show that 6 wk of DOCA-induced hypertension in mice causes podocyte damage, including lower canonical gene expression, reduced podocyte density, and reduced expression of both canonical podocyte genes as well as genes coding for proteins key to glomerular function, such as Vegfa.

### High-Fat Diet Induces an Inflammatory Podocyte Phenotype

Next, we compared the impact of hypertension on podocyte health with the effects of obesity by providing 4-mo-old mice with an ad libitum HFD for 12 wk. Control mice fed a normal diet for 12 wk had an initial average body weight of 29.9 ± 1.7 g at 4 mo of age that increased by 17% to 35.1 ± 1.3 g (*P* < 0.0001) (Supplemental Fig. S5*A*). For reference, 6-mo-old male C57BL/6J mice had average body weight of 30.7 ± 2.0 g, according to The Jackson Laboratory Mouse Phenome Database (44). In the separate group of mice given a HFD, their initial weights at 4 mo of age were 30.4 ± 2.4 g. HFD-fed mice gained an average weekly increase of 3.9 ± 2.2% in body weight ending at a final average weight of 48.5 ± 6.5 g or a 58.4% increase from their initial weight (*P* < 0.0001) (Supplemental Fig. S5*B*). Furthermore, oil red O staining demonstrated increased lipid accumulation in the glomeruli of HFD-fed mice (Supplemental Fig. S5, *C* and *D*).

**Figure 4.**
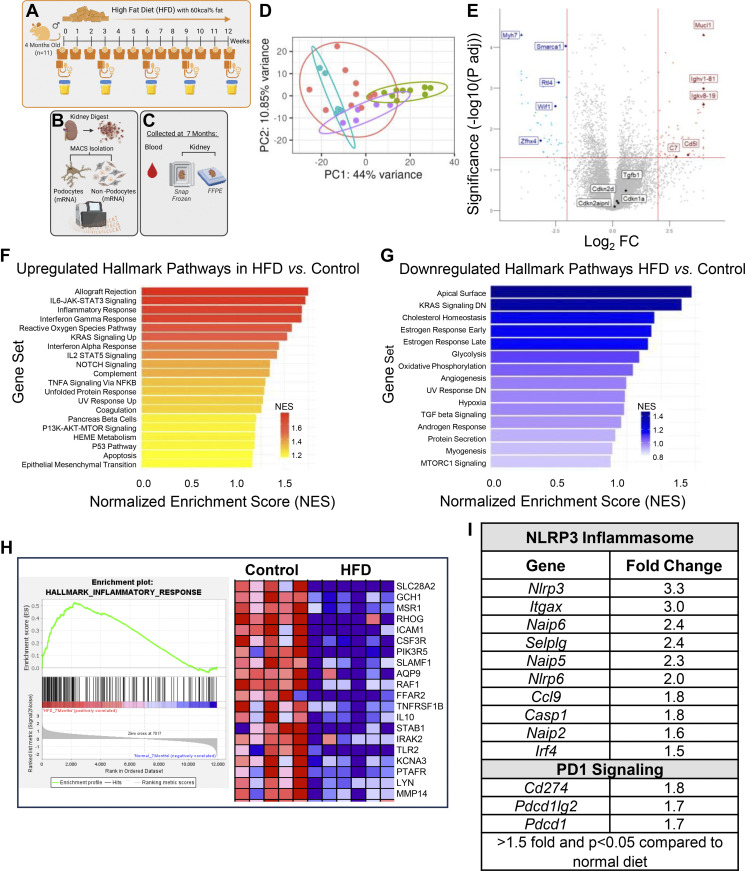
Inflammatory pathways are increased in podocytes in mice treated with a high-fat diet (HFD). *A*–*C*: schema of experimental protocol for HFD in mice. Following a HFD for 12 wk (*A*), podocytes isolated by magnetic-activated cell sorting (MACS) underwent bulk RNA-sequencing (*B*) and kidneys were collected and fixed for immunostaining (*C*). *D*: principal component analysis (PCA). There is no overlap between HFD mice (purple) and their controls (blue). Shown too are the PCA for mice on deoxycorticosterone acetate (DOCA, green) and their controls (red). The latter are a different age to controls for HFD. The PCA shows no overlap between DOCA and HFD mice. *E*: volcano plot shows genes positively and negatively enriched by HFD. *F*–*H*: Hallmark pathway analyses. *F*: inflammatory pathways are the predominant pathways upregulated by HFD, with examples of the top 20 genes shown for the Hallmark inflammatory response pathway (*H*). *G*: downregulated Hallmark pathways include several metabolic processes. *I*: genes in the NLR family pyrin domain containing 3 (NLRP3) and PD1 signaling pathways are increased in podocytes of HFD mice.

As with DOCA-treated mice, we isolated podocytes and performed an unbiased analysis of transcriptomic changes comparing podocytes from obese versus non-obese mice (Fig. 4*B*). PCA analysis and volcano plot comparing the two conditions (control vs. HFD) again identified dramatic differences (Fig. 4*D*). Moreover, there was no overlap between mice on HFD and mice treated with DOCA (Fig. 4*D*). The volcano plot shows genes positively and negatively enriched by HFD (Fig. 4*E*). Hallmark pathway GSEA analysis showed that several inflammatory-associated pathways were enriched in podocytes from HFD mice, such as allograft rejection, IL-6-JAK-Stat signaling, inflammatory response, IFN-γ and IFN-α, reactive oxygen species, IL-2-Stat5, p53 pathway (Fig. 4, *F* and *H*), and epithelial-mesenchymal transition (Fig. 4*F* and Supplemental Fig. S6, *A* and *B*). Conversely, Hallmark pathways downregulated in podocytes from obese mice included apical proteins, KRAS signaling, cholesterol homeostasis, and oxidative phosphorylation (Fig. 4*G* and Supplemental Fig. S6, *C* and *D*). Noteworthy increases were genes of the NLRP3 inflammasome (e.g., *Nlrp3* and its downstream mediator *Casp1*) and the PD-1 signaling pathway (e.g., *Cd274*, *Pdcd1lg2*, and *Pdcd1*) (Fig. 4*I*).

### A Senescent and Aged Phenotype Accompanies High-Fat Diet-Induced Obesity in Mice

Next, we investigated whether obesity results in premature aging and senescence in mice on a 12-wk HFD. Analysis of the FRIDMAN_SENESCENCE_UP (45), cellular senescence Reactome (46), and RODWELL_AGING_KIDNEY_UP (40) gene sets were enriched in obese versus nonobese mice (Table 4). Examples include increased C-C motif chemokine ligand 5 (*Ccl5*), glycoprotein nonmetastatic melanoma protein b (*Gpnmb*), a marker of tubular injury (47), and stimulator of interferon genes (*Sting1*), a gene that has a key role in APOL1-associated podocytopathy (48).

**Table 4.** Senescent and aging genes in podocytes of HFD mice

	Gene Name	*Fold Change in HFD vs. Controls
FRIDMAN_SENESCENCE_UP Gene List	*Hsp5*	1.5
	*Optn*	−1.5
Senescence Reactome	*Mdm4*	1.7
	*Mapkl4*	1.6
	*Eed*	1.6
	*Mink1*	1.5
RODWELL_AGING_KIDNEY_UP Gene List	*C7*	7.0
	*Gpnmb*	5.7
	*Jchain*	4.9
	*Igkc*	4.4
	*Ms4a7*	4.3
	*Lcp2*	3.2
	*Slc15a3*	2.9
	*Mpeg1*	2.9
	*Ccl5*	2.7
	*Col27a1*	2.7
	*Vcam1*	2.7
	*Cxcl16*	2.5
	*Grn*	2.3
	*Birc3*	2.3
	*Coro7*	2.2
	*Arhgef6*	2.2
	*Irak2*	2.1
	*Sting1*	2.0
	*Man2b1*	1.9
	*Prag1*	1.6
	*Syngr2*	1.6
	*Coro1c*	1.5
	*Zcchc7*	1.5
	*Dlgap1*	−3.8
	*Lix1*	−3.0
	*Tspan1*	−2.3
	*Tesc*	−2.2
	*Wfdc2*	−2.1
	*Tspan18*	−1.9
	*Cdo1*	−1.8
	*Gpx8*	−1.7
	*Aebp1*	−1.7

HFD, high-fat diet.

*Defined as >1.5-fold and *P* value < 0.05.

**Figure 5.**
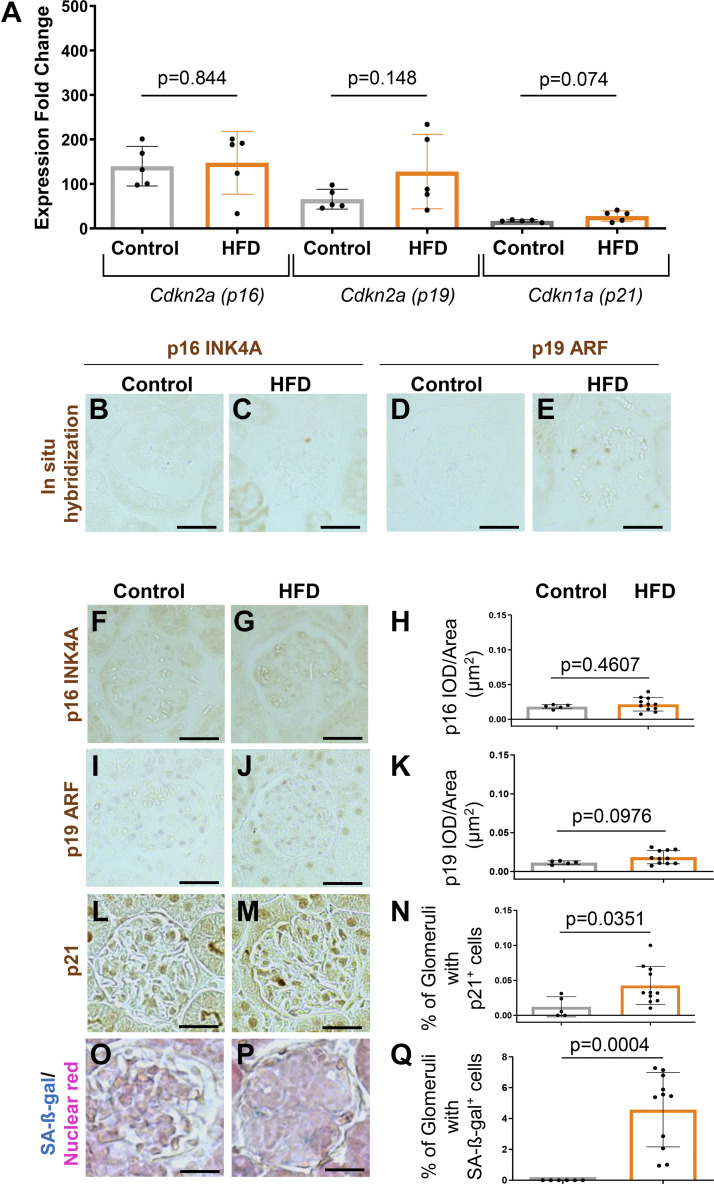
High-fat diet (HFD) induced obesity in young mice is accompanied by stress-induced senescence. *A*: qRT-PCR on isolated podocytes. There were no differences in the expression for *Cdkn2a* (p16 INK4A, variant 2), *Cdkn2a* (p19 ARF, variant 1), and *Cdkn1a* (p21) between control and HFD mice. *B*–*E*: in situ hybridization. Neither *Cdkn2a* (p16 INK4A, variant 2) (*B* and *C*) nor *Cdkn2a* (p19 ARF, variant 1) (*D* and *E*) increased in glomeruli of HFD mice. *F*–*N*: immunoperoxidase staining (brown) and quantitation. Immunostaining for p16 (*F*–*H*) and p19 (*I*–*K*) did not change in mice given a HFD. In contrast, staining for p21 was higher in HFD-treated mice, accompanied by an increase in the senescent maker SA-β-galactosidase (SA-β-gal) (blue) (*O*–*Q*). Scale bar: 40 μm.

In contrast to DOCA-treated hypertensive mice, the aging and senescence phenotype was not accompanied by statistically significant changes in the age-inducing senescent genes *Cdkn2a,* variant 2 (p16 INK4A), or *Cdkn2a,* variant 1 (p19 ARF), as measured by qRT-PCR (Fig. 5*A*), in situ hybridization (Fig. 5, *B–E*), or immunostaining (Fig. 5, *F–K*). However, immunostaining for the DNA repair pathway component *Cdkn1a* (p21) was increased (Fig. 5, *L*–*N*) as well as glomerular staining for the senescent marker SA-β-gal (Fig. 5, *O*–*Q*) in mice fed HFD compared with age-matched mice on a normal diet. Together, these results show that in obese mice, podocytes express markers consistent with a stress-induced senescence (p21, p53, and SA-β-gal-positive) phenotype.

### High-Fat Diet Lowers Podocyte Canonical Gene Expression and Podocyte Density

**Figure 6.**
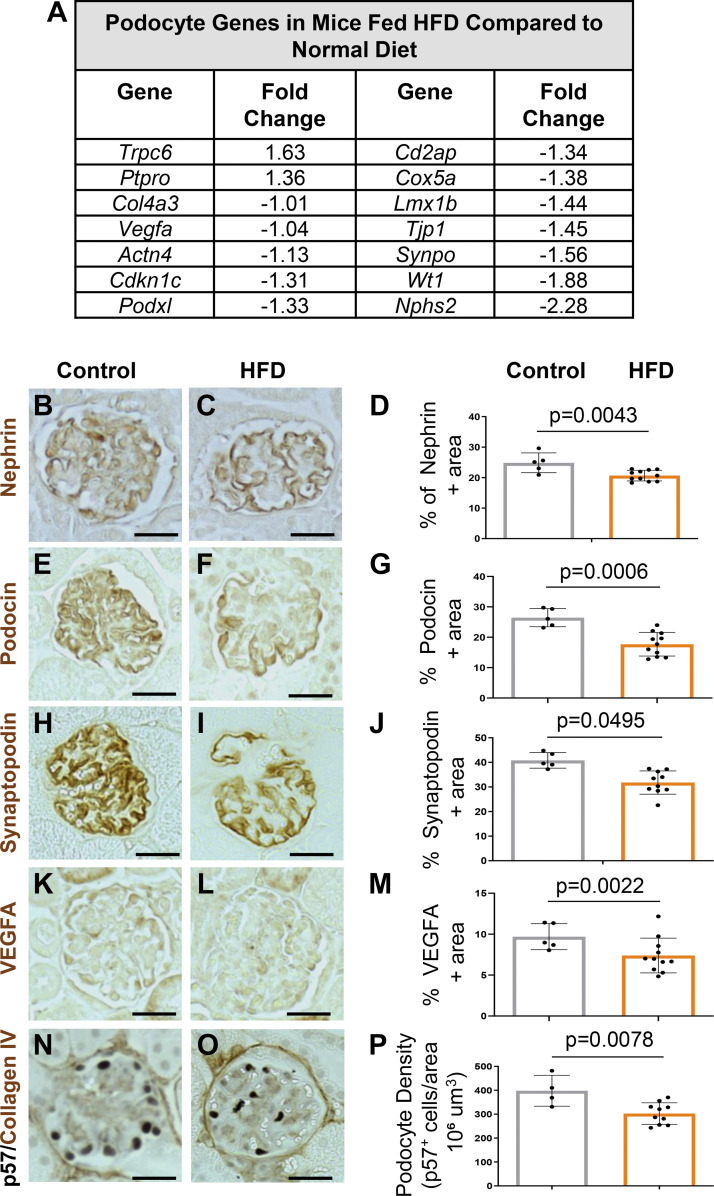
Canonical podocyte genes are lower in obese mice fed a high-fat diet (HFD). *A*: RNA-sequencing. Table shows fold change decreases in gene expression for canonical podocyte genes in HFD-fed mice compared with controls. *B*–*P*: immunoperoxidase staining (brown) and quantitation. Compared with controls staining was lower in HFD-fed mice for nephrin (*B*–*D*), podocin (*E*–*G*), synaptopodin (*H* and *J*), VEGFa (*K*–*M*), and p57 (*N*–*P*).

Finally, we compared canonical podocyte gene expression between obese and nonobese mice. Indeed, the RNA-sequencing data demonstrated reduced transcript levels of many canonical genes such as *Nphs2*, *Wt1*, and *Synpo* (Fig. 6*A*). This was accompanied by reduced immunostaining for nephrin, podocin, synaptopodin, and VEGFa (Fig. 6, *B–M*). Interestingly, similar to DOCA-treated mice, *Ptpro/*Glepp-1 was increased in HFD-fed mice compared with controls and was also accompanied by increased levels of transient receptor potential cation channel 6 (*Trpc6*) (Fig. 6*A*). Finally, the density of podocytes was significantly lower in mice on a HFD diet compared with normal diet-fed mice (398 ± 32 vs. 302 ± 14 p57^+^ cells/10^6^ μm^3^, *P* = 0.008) (Fig. 6, *N–P*).

## DISCUSSION

DOCA and HFD treatments are well-established translational mouse models to study the pathophysiology and mechanisms of hypertension and obesity, respectively. Indeed, studies have shown that, like humans, they cause podocyte injury (12–14, 20, 23, 49). Our results confirmed the presence of podocyte injury in both models. This included a decrease in podocyte density, a reduction in the expression of key canonical podocyte genes and reduced expression of genes encoding proteins that are essential for glomerular maintenance and function, e.g., *Vegfa*. Nevertheless, the current study now extends on these findings. To our knowledge, we show for the first time that in young mice, a short duration of hypertension following DOCA administration for 6 wk or obesity upon feeding a HFD for 12 wk leads to the de novo expression of markers of inflammation, senescence, and aging and the establishment of a SASP in podocytes. To better understand the transcriptional changes associated with these phenotypes, we performed unbiased bulk RNA sequencing. In addition to the induction of genes representing senescence, a SASP, and aging, we observed a differential profile of senescence-inducing genes. Although podocytes from young mice treated with DOCA-salt exhibited expression of age-inducing senescent genes *Cdkn2a* (p16 INK4A, variant 2) and *Cdkn2a* (p19 ARF, variant 1), as well as stress-inducing senescent gene *Cdkn1a* (p21) and the *Tp53* and DNA damage repair pathways, mice that developed obesity by feeding HFD showed increased expression/activation of *Cdkn1a* (p21), as well as the* Tp53* and DNA damage repair pathways, but not age-inducing senescent genes *Cdkn2a* (p16 INK4A, variant 2) and *Cdkn2a* (p19 ARF, variant 1). In the context of an increase in the senescence marker SA-β-gal in young mice with hypertension or obesity, the increase in these senescent inducing genes in young mice suggests that they are likely causing the senescent/aged phenotype in podocytes.

Senescent proteins p16 and p19 typically cause cellular senescence in healthy aging (50). In fact, we and others have reported an increase in p16 and p19 in middle-aged and aged glomerular cells, including podocytes, and in aged tubular epithelial cells (27, 33, 35, 51, 52). More recently, we reported an increase in p16 following acute podocyte injury in an experimental model of FSGS (53). Conversely, p21 and p53 typically cause stress-induced senescence (but can contribute to age-associated senescence). Studies have shown expression of p21 and p53 in aged podocytes (27, 52) and p21 and p53 are also increased following stress-induced senescence in podocytes (54, 55), including podocytes injured by complement (56). In podocytes, p21 is required for TGF-β1-induced apoptosis (57) and limits proliferation in cultured podocytes exposed to mechanical stretch (58). In contrast, to a recent study (55), the stress-induced podocytes in the current study did not exhibit changes in the expression of HDAC1 and 2. Thus, our results are consistent with experimental hypertension and obesity being associated with the de novo development of a podocyte senescent and aged phenotype in mice. Yet, there are differences between hypertension and obesity-associated podocyte senescence. Both are stress-induced, but the one in hypertensive mice is superimposed by age-associated senescence form. The consequences of this potentially accelerated podocyte/glomerular aging during hypertension or obesity needs further study.

It is important to note that the connection between obesity and senescence has been reported in other organs. For example, obesity induces senescence in the glial cells of the lateral ventricle in mice (59) and increases p16 and p21 levels in the livers of rats (60). Moreover, cellular senescence has been implicated as a causal factor in obesity-related inflammation (61). In mice fed a HFD that developed obesity, renal tubular cells began to express the senescent markers p16 and p53 that were accompanied by a SASP phenotype (62).

It is interesting to note that endothelial senescence has been implicated in the pathogenesis of systemic hypertension (for a review, see Ref. 63) and pulmonary hypertension (64). Surprisingly, no published data show that systemic hypertension causes endothelial senescence. Similarly, we did not detect changes in the glomerular endothelial marker gene expression in the hypertensive mice in the current study.

As expected for a podocyte injury model, podocyte density was reduced in both mouse models compared with their age-matched controls. Although we have not directly investigated the underlying mechanism, several possible mechanisms for podocyte loss include apoptosis, pyroptosis, and p53 pathways, all of which were increased in our two mouse models. Among the highest enriched GSEA pathways in podocytes from hypertensive and obese mice were several related to inflammation. Increased NLRP3 activity has been reported in podocytes following HFD feeding (23). We have recently reported on the importance of PD-1 and NLRP3-inflammasome signaling (33, 35). Both pathways are increased in middle-aged and aged podocytes and under experimental FSGS conditions (53). Moreover, inhibiting either of these two forms of intracellular inflammation in middle-aged mice reduced the senescence/aged phenotype. Importantly, this led to a longer podocyte lifespan and improved health span.

In addition, the GSEA identified that the reactive oxygen species gene set is enriched, and the oxidative phosphorylation gene set is depleted in podocytes of obese mice. This agrees with the observation that oxidative stress has been reported in podocytes in mice with HFD (49). Moreover, administering the mitochondrial stabilizer SS-31 prevented the loss of podocytes in mice fed HFD, suggesting abnormal mitochondrial bioenergetics contribute to podocyte dysfunction/loss in obese mice (65). Finally, several canonical podocyte genes were decreased in DOCA-treated and HFD-fed mice. These included structural podocyte proteins such as nephrin, podocin, and synaptopodin, as well as VEGFa, which is often used as a proxy for podocyte function (66). It is interesting that podocyte damage preceded any damage to glomerular endothelial cells or activation of parietal epithelial cells within the glomerulus, as markers for these were not changed.

We acknowledge several limitations of the study. The study aimed to determine how these common clinical stressors (hypertension and obesity) impact podocytes qualitatively, but not to compare their injury responses quantitatively. Although we did not provide mechanisms for the inflammation/senescent/aging changes in injured podocytes, nor the other transcriptional changes, this body of work provides a wealth of information for future studies, including several candidate genes that may warrant in-depth analysis. For example, *Mmp7,* a downstream target of β-catenin, has been previously shown to be secreted from tubules, decrease nephrin levels, and increase proteinuria (67). Similarly, *Prg4*, a ligand for CD44 (68), is increased in parietal epithelial cells following podocyte injury and contributes to the fibrotic response during glomerular injury (69). Strain considerations are always important, as has been reported in mice on a HFD (70). Finally, our study was limited to male mice. Male mice were chosen for this study as hypertension and renal disease tend to be less severe in female mice due to the protective effects of estrogen (71). Comparable future studies in female mice should be considered. Although we have not been able to detect sex differences in podocytes during aging (27), the emerging data on sex-specific immune responses in a wide range of diseases warrants a similar comparative analysis for podocyte injury.

In summary, this study provides tantalizing data on how a short period of hypertension or obesity can dramatically alter podocyte biology. In particular, the long-term implications and consequences of premature podocyte senescence and aging are a so far unrealized paradigm of glomerular biology. However, they will likely be a critical aspect to understand kidney health in the future.

## DATA AVAILABILITY

The raw data supporting the conclusions of this article will be made available upon reasonable request.

## SUPPLEMENTAL DATA

10.6084/m9.figshare.25236832.v1 Supplemental Figs. S1–S6 and Supplemental Table S1: https://doi.org/10.6084/m9.figshare.25236832.v1.

## GRANTS

S.J.S. and O.W. were supported by National Institute of Diabetes and Digestive and Kidney Diseases Grants 5R01DK056799-10, 5R01DK056799-12, 1R01DK097598-01A1, UC2DK126006-2, and 1R01DK090358-12 and by Department of Defense Grant DOD PR180585/PR180585P1. T.C. was supported by National Institute of Diabetes and Digestive and Kidney Diseases Grants R01DK127634 and RC2DK125960 and by Cancer Prevention and Research Institute of Texas Grant RP220201.

## DISCLOSURES

No conflicts of interest, financial or otherwise, are declared by the authors.

## AUTHOR CONTRIBUTIONS

D.G.E., J.W.P., O.W., and S.J.S. conceived and designed research; S.R.M., N.K., R.A.S., C.C., D.G.E., and J.W.P. performed experiments; S.R.M., N.K., R.A.S., C.C., D.G.E., B.M.V.P., B.K., J.W.P., O.W., and S.J.S. analyzed data; S.R.M., N.K., R.A.S., C.C., D.G.E., J.W.P., O.W., and S.J.S. interpreted results of experiments; S.R.M., N.K., D.G.E., J.W.P., and S.J.S. prepared figures; S.R.M., N.K., J.W.P., O.W., and S.J.S. drafted manuscript; S.R.M., N.K., D.G.E., J.W.P., O.W., and S.J.S. edited and revised manuscript; S.R.M., N.K., J.W.P., O.W., and S.J.S. approved final version of manuscript.
